# Induced Abortion as a Family Planning Strategy in Romania: A Narrative Literature Review of Policy, Health Outcomes and Psychosocial Determinants

**DOI:** 10.3390/healthcare14203372

**Published:** 2026-10-09

**Authors:** Fernanda-Ecaterina Augustin, Bashar Haj Hamoud, Liana Ples, Mircea-Octavian Poenaru, Gabriel-Petre Gorecki, Mihai-Daniel Dinu, Adrian Neacşu, Romina-Marina Sima

**Affiliations:** 1 Department PhD, IOSUD-Institution Offering Doctoral Studies, “Carol Davila” University of Medicine and Pharmacy, 020021 Bucharest, Romania; fernanda-ecaterina.augustin@drd.umfcd.ro; 2 Department of Obstetrics and Gynecology, “Carol Davila” University of Medicine and Pharmacy, 020021 Bucharest, Romania; liana.ples@umfcd.ro (L.P.); mircea.poenaru@umfcd.ro (M.-O.P.); adrianneacsu2006@yahoo.com (A.N.); romina.sima@umfcd.ro (R.-M.S.); 3 Department of Obstetrics and Gynecology, “Bucur” Maternity, Saint John Hospital, 040294 Bucharest, Romania; 4 Department for Gynecology, Obstetrics and Reproductive Medicine, Saarland University Hospital, Kirrberger Straße 100, Building 9, 66421 Homburg, Germany; bashar.hajhamoud@uks.eu; 5 Department of Anesthesia and Intensive Care, Faculty of Medicine, “Titu Maiorescu” University, 031593 Bucharest, Romania; gabriel.gorecki@prof.utm.ro; 6 Department of Anesthesia and Intensive Care, CF2 Clinical Hospital, 011464 Bucharest, Romania

**Keywords:** induced abortion, family planning services, maternal mortality, contraceptive access, Romania, abortion stigma

## Abstract

**Highlights:**

**What are the main findings?**

- Romania’s experience shows that restrictive abortion policies and limited contraceptive access were associated with unsafe abortion and high maternal mortality, whereas legal access combined with family-planning programs coincided with major reductions in abortion-related deaths and abortion rates.
- Decisions regarding induced abortion are shaped by intersecting socioeconomic, relational, educational, and reproductive-life-course factors; long-term psychological outcomes are more strongly associated with pre-existing mental-health vulnerability, social support, stigma, and decisional autonomy than with abortion itself.

**What are the implications of the main findings?**

- Reproductive-health policies should integrate equitable access to effective contraception, comprehensive sexual education, safe abortion care, and post-abortion counselling to prevent unintended pregnancy and avoidable maternal morbidity.
- Clinicians should provide person-centered, nonjudgmental counselling that respects autonomy, assesses individual psychosocial support needs, and uses neutral terminology—such as “induced abortion”—to reduce stigma in care and scientific communication.

**Abstract:**

**Background**: Induced abortion is a major component of reproductive health, with about one in four pregnancies worldwide ending in induced abortion and important implications for maternal morbidity and mortality. Romania’s history of restrictive pronatalist policies, very high post-1989 abortion rates, and subsequent family planning reforms provides an informative case for understanding abortion as a family planning strategy. **Objectives**: This narrative literature review synthesizes evidence on induced abortion with emphasis on Romania. It describes the evolution of abortion law and policy, summarizes epidemiological and maternal health outcomes, examines sociodemographic and psychosocial determinants of induced abortion, reviews psychological and psycho-sexual consequences, and discusses the role of non-stigmatizing terminology in clinical communication. **Methods**: This narrative literature review was informed by a structured literature search of PubMed, Scopus, and Web of Science Core Collection conducted on 20 May 2026. Publications from January 1990 to September 2024 were searched using terms related to induced abortion, family planning, Romania, maternal mortality and morbidity, abortion policy, psychosocial determinants, psychological outcomes, and stigma. Predefined eligibility criteria were used to identify relevant peer-reviewed studies and major institutional sources; additional historical and legal documents were considered for contextual framing. Additional recently published studies relevant to the review themes were identified through targeted update searches and reference-list screening up to 20 May 2026. **Results**: Restrictive policies and contraceptive bans in Romania were associated with extremely high abortion-related maternal mortality, whereas legal liberalization and integrated family planning programs led to marked declines in abortion rates and deaths. Abortion decisions reflect age, partnership, parity, education, employment and legal status. Psychological studies and major reviews report heterogeneous post-abortion experiences. These outcomes appear to be influenced primarily by pre-existing mental-health vulnerability, pregnancy intention and circumstances, social support, stigma, and decisional autonomy, rather than by the procedure alone. Also, terminology and conscientious objection practices can contribute to stigma and access barriers. **Conclusions**: Ensuring equitable access to contraception, safe abortion services, nonjudgmental counseling and neutral professional language appears essential for reducing preventable maternal morbidity and supporting reproductive autonomy.

## 1. Introduction

Induced abortion remains a major component of reproductive health worldwide, with an estimated one in four pregnancies ending in induced abortion and important implications for maternal morbidity and mortality [1]. In Romania, abortion has played a central role in family planning practices, shaped by decades of restrictive pronatalist policies followed by rapid liberalization after 1989 [1]. Romania’s history of restrictive pronatalist policy, very high post-1989 abortion rates, and subsequent family planning reforms makes it a particularly informative case for understanding abortion as a family planning strategy. In this review, the term “abortion as a family planning strategy” does not imply that induced abortion is equivalent to contraception or should be considered a contraceptive method. Rather, it describes the historical and social use of abortion by some women or couples to manage unintended pregnancy, regulate the timing or number of births, or limit childbearing in settings characterized by limited access to effective contraception, sexual education, and reproductive-health services.

Despite this history, comprehensive national data on abortion as a method of family planning are still lacking, because reporting is not uniformly required across all public and private healthcare facilities. This gap limits realistic assessment of abortion patterns and their determinants, and it highlights the need for integrative analyses that connect legislative evolution, service organization, contraceptive access, and women’s lived experiences. Existing publications often address these dimensions separately, so there is limited integrative work that connects legal frameworks, service provision, contraceptive uptake, psychosocial determinants, and psychological outcomes in a single narrative.

In this narrative literature review, we synthesize current evidence on induced abortion as a family planning strategy, with particular emphasis on the Romanian context. We examine the historical and legislative background of abortion, epidemiology and health outcomes related to unsafe and legal abortions, sociodemographic and psychosocial determinants of induced abortion, its psychological consequences, and the role of non-discriminatory terminology in clinical and scientific communication. The overarching aim is to provide a clinically and socially contextualized understanding that can inform counseling, public health interventions, and reproductive health policy. The review is intended for clinicians, public health professionals, and policymakers involved in reproductive health, particularly in settings undergoing legislative or service reforms.

## 2. Materials and Methods

This study was designed as a narrative literature review informed by a structured literature search. A narrative approach was selected because the objective was to integrate heterogeneous evidence concerning the historical, legal, epidemiological, policy, clinical, psychosocial, and terminology-related dimensions of induced abortion, with particular emphasis on Romania. The structured search and predefined eligibility criteria were used to improve transparency, breadth, and consistency in source identification and selection.

Searches were conducted in PubMed, Scopus, and Web of Science Core Collection on 20 May 2026. The primary search covered publications from 1 January 1990 to 30 September 2024. To identify more recently published literature, targeted update searches were conducted on 20 May 2026 using the same core search concepts and covering the period from 1 October 2024 to 20 May 2026. Reference lists of key reviews, institutional reports, policy documents, and relevant articles were also screened to identify additional sources. The search strategy included terms related to induced abortion, voluntary termination of pregnancy, unsafe abortion, family planning, contraception, Romania, abortion law and policy, maternal mortality and morbidity, post-abortion contraception, sociodemographic determinants, psychological outcomes, stigma, terminology, and conscientious objection.

Eligible sources included peer-reviewed publications and major institutional reports relevant to at least one of the review domains: abortion incidence and trends; maternal mortality or morbidity attributable to abortion; legal and policy changes affecting access; family planning and contraceptive services; sociodemographic or psychosocial determinants of abortion decisions; psychological outcomes after induced abortion; and terminology or stigma in abortion care. Classic studies from the late communist and early post-communist period, as well as selected national and institutional documents, were also considered when they provided essential historical, legal, policy, or statistical context for Romania.

Exclusion criteria involved non-peer-reviewed publications, conference abstracts, case reports or very small series (for example, fewer than 20 participants), studies lacking sufficient clinical, epidemiological, or methodological detail, and articles outside the defined timeframe when they did not provide unique historical information. Non-English publications were excluded unless they contained essential national data that could not be obtained from other sources. Legal, historical, and ethical documents without empirical data were not included as core evidence sources; however, selected sources were retained to provide essential historical, legislative, and institutional context. Given the heterogeneous nature of the included sources and the narrative purpose of the review, no formal study-level risk-of-bias assessment or certainty-of-evidence grading was undertaken. A single standardized appraisal tool was not considered appropriate for the combination of observational and quasi-experimental studies, qualitative research, reviews, institutional reports, statistical sources, and historical or legal documents included in this review. Instead, sources were considered according to their relevance to the predefined review domains, adequacy of methodological or descriptive reporting, publication source, consistency with other available evidence, and contribution to the Romanian historical, legal, epidemiological, clinical, or psychosocial context. Priority was given to peer-reviewed research and reports from recognized national or international institutions.

The electronic search across PubMed, Scopus, and Web of Science yielded a little over 200 records. After title and abstract screening for relevance to induced abortion, family planning, and women’s health, with particular emphasis on Romania and comparative international evidence, approximately 60 full-text articles and reports were assessed. A total of 45 sources were retained and cited in the narrative review, including original empirical studies, reviews and evidence syntheses, institutional reports, statistical sources, and historical or legal contextual documents. The classification and principal role of the included sources are reported in Supplementary Table S1. Sources published in 2025 and 2026 were identified through the targeted update searches and/or reference-list screening and were assessed according to the same relevance-based eligibility criteria used for sources identified through the primary search.

Titles and abstracts were screened for relevance to the predefined review domains, and potentially relevant full-text records were assessed against the eligibility criteria. Where eligibility, source classification, or interpretation was uncertain, the issue was resolved through discussion among the authors. Evidence was synthesized narratively and organized according to five predefined domains: historical and legislative context; epidemiology and maternal health outcomes; sociodemographic and psychosocial determinants; psychological and psychosexual outcomes; and terminology, stigma, and access to care. Findings were compared across source types and settings, with particular attention to the Romanian context. Where findings were heterogeneous or conflicting, this variability was described and interpreted in relation to differences in study design, population, setting, outcome measures, and contextual factors; no quantitative pooling was undertaken.

Reference lists of key reviews, institutional reports, and policy documents were also screened to identify additional sources relevant to the Romanian context. Of these 45 sources, 37 contributed directly to the evidence synthesis, including empirical studies, systematic or narrative reviews, and institutional/statistical sources reporting epidemiological, clinical, psychosocial, or program-related data. The remaining 8 sources were used primarily to establish the Romanian historical, legal, and institutional context, including Decree No. 770, historical publications on the pronatalist period, and legal or policy documents. The source classification was based on the principal function of each reference in the present review; some institutional documents also contributed descriptive data relevant to the synthesis. The selection process was intended to support a comprehensive and transparent interpretive synthesis of heterogeneous literature, rather than to establish an exhaustive systematic-review dataset.

Reference lists of key WHO reports on unsafe abortion, APA and NCCMH reviews of mental-health outcomes after abortion, and strategic assessments of abortion and contraception in Romania were cross-checked to identify additional relevant evidence and contextual sources. No restriction on study design was applied beyond clinical and public-health relevance; observational studies, quasi-experimental analyses, randomized trials, qualitative research, reviews, and major institutional reports were considered (Figure 1). Classification and role of the 45 sources cited in the narrative review are reported in Supplementary Table S1. Database-specific search concepts, search terms, limits, and dates are reported in Supplementary Table S2.

As a narrative review, this work may be subject to selection and interpretive bias. To enhance transparency, we used predefined review domains and eligibility criteria, conducted structured searches in three bibliographic databases, screened titles, abstracts, and full texts, and screened reference lists of key sources. Nevertheless, no formal systematic-review or scoping-review reporting framework, risk-of-bias tool, certainty-of-evidence assessment, or meta-analysis was applied. Figure 1 is included to transparently summarize the structured identification and selection of sources and is not intended to represent a PRISMA-based systematic-review flow diagram. Because this study was based exclusively on published and publicly available sources and involved no new human participant data, institutional review board approval was not required.

## 3. Narrative Review of the Literature

This section brings together the main directions in the literature regarding induced abortion, analyzing its evolution in Romania, the frequent justifications for resorting to this procedure, and the impact of legislation on maternal health. The consequences of unsafe abortions, preventive measures through access to contraception, and the psychological aspects associated with abortion are highlighted. The role of medical language in shaping perceptions of this topic is also discussed. This narrative literature review provides the theoretical foundations for a comprehensive understanding of the phenomenon in the current socioeconomic context.

In this review, induced abortion is used as the preferred neutral clinical term for deliberate termination of pregnancy. Voluntary termination of pregnancy (VTP) is retained only when reporting terminology used in cited studies; where relevant, termination of pregnancy on request refers to an induced abortion requested by the pregnant person within the applicable legal framework. The term elective abortion is avoided because it may imply that the decision is optional or clinically insignificant and can reinforce moral hierarchies.

### 3.1. Historical and Legislative Background

In October 1966, the communist regime led by Nicolae Ceaușescu introduced one of the most restrictive pronatalist policies in Europe, prohibiting abortion in almost all circumstances. Exceptions were limited to special cases, such as women over the age of 45 with at least four children, pregnancies resulting from rape or incest, serious risks to the mother’s life, or the diagnosis of congenital fetal malformations [2]. This policy materialized through the tightening of abortion legislation, concretized in two major stages—1966 and 1985—and through the prohibition of modern contraceptive methods [3,4,5,6,7,8]. The effects of this restrictive orientation on maternal health were considerable: in 1989, Romania recorded the highest maternal mortality rate in Europe, with 170 maternal deaths per 100,000 live births, of which 87% were attributed to complications resulting from unsafe abortions [4,9].

After the fall of the communist regime in December 1989, the new Romanian government immediately abolished the restrictive legislation concerning abortion [4]. Between 1990 and 1992, Romania recorded the highest abortion rate in the world, with an alarming figure of 182 abortions per 1000 women of reproductive age, reflecting both the lack of access to modern contraceptive methods and the post-revolution socioeconomic transformations [10]. Subsequently, in 1996, legislation was adopted regulating women’s right to request induced abortion during the first 12 weeks of gestation. Beyond this period, abortion is permitted only in situations in which it is necessary to protect the life or physical integrity of the woman. The procedure may be performed exclusively by a gynecologist in an authorized healthcare facility [11].

Data on legal abortions in Romania for 1990–1992 were retrieved from the INSSE TEMPO-Online database [10]. In 1991, 789,096 legal abortions were recorded, while the number of births was approximately 270,000. This disproportion—approximately three abortions for every birth—reflects the limited availability of effective contraception and reproductive-health services during the early post-communist transition [10,12], (Table 1).

A study conducted in Romania after 1989, immediately following the legalization of abortion, brought to the forefront the perspective of women of reproductive age in a context profoundly affected by the post-communist transition [13]. The research included 1000 women who were either about to undergo or had already undergone an induced abortion. In parallel, gynecologists involved in performing these procedures were also interviewed, with the aim of capturing both the perspectives of the patients and of medical professionals [13].

According to the study results, most respondents considered the use of a contraceptive method to be much more accessible and less costly, both physically and emotionally, than abortion. In addition, 84% of the women interviewed expressed their intention to adopt a contraceptive method in the future [13]. However, the socioeconomic reality of the early 1990s profoundly influenced the actual capacity for family planning. Many contraceptive methods were unavailable, and sexual education was practically nonexistent in the public sphere and within educational institutions.

The responses of gynecologists brought into discussion a series of systemic issues affecting contraceptive counseling. Most indicated that women expected the physician to initiate the discussion about contraception in 69% of cases. Nevertheless, some of them regarded contraceptive counseling as a marginal activity, below their professional level, citing high workload, lack of resources, and low remuneration during that period. One physician pointed out that the time required to provide counseling to a single patient could have been used to perform four or five procedures, suggesting a systemic pressure oriented more toward invasive interventions than toward prevention [13].

Legislative reform was complemented by a series of initiatives designed to improve access to family planning and reproductive health services. Thus, with the support of a World Bank loan, the Ministry of Health established by 1994 a total of 11 family planning reference centers and 230 specialized offices throughout the country [11,14]. At the same time, a Sexual Education and Family Planning Unit was created, responsible for the procurement of contraceptives, the development and implementation of national information campaigns, the elaboration of training programs for medical students and nurses, and the training of physicians in contraceptive counseling [3,11]. Funding provided by the World Bank also enabled the acquisition of modern equipment for abortion procedures, such as electric aspirators. However, in the absence of a dedicated maintenance budget, medical personnel in some cases returned to traditional methods that were more invasive and carried higher risk, such as dilatation and curettage [3].

In 2001, with the support of USAID and John Snow Inc., the Romanian Family Health Initiative (RFHI) was launched. This initiative aimed to expand access to family planning and reproductive health services, especially among vulnerable populations. Among the most important achievements of RFHI was the modification of the legislative framework so that family physicians received responsibilities in the provision of contraceptive services, which had previously been reserved exclusively for gynecologists. The program also supported the integration of reproductive health services into the primary healthcare network [3,11].

Within RFHI, the National Family Planning Program (NFPP) was also implemented, structured along three major lines of action. The first component targeted the professional training of family physicians and nurses, resulting in the mobilization of 5105 physicians and 3063 nurses in the provision of contraceptive services by 2006 [11]. The second line of action involved mass communication campaigns aimed at increasing demand for contraceptive methods among Romanian women, who until then had frequently used abortion as the main method of family planning [3,11,15,16]. The final component of the program was directed toward improving contraceptive accessibility through significant cost reductions for vulnerable social groups [11,15,17]. During the final implementation period (2005–2007), RFHI provided medical training on post-abortion and postpartum contraceptive counseling in 52 public obstetrics and gynecology clinics, which together accounted for more than half of all abortions performed nationally [11].

As a result of these coordinated interventions, the use of modern contraceptive methods increased significantly: from 13.9% in 1993 to 29.5% in 1999 and 38.2% in 2004. Particularly notable increases were observed in rural areas, where the prevalence of modern contraceptive use rose from 20.9% in 1999 to 33.0% in 2004 during the RFHI program period [11,18,19]. In parallel, the abortion rate declined substantially, reaching 27.1 per 1000 women in 2006 [12].

At the same time, statistical data indicate a spectacular decrease in maternal mortality associated with unsafe abortions after 1989. The annual maternal mortality rate caused by abortion fell from 148 deaths per 100,000 live births in 1989 to 58 in the following year [4,9]. Whereas in 1989, 87% of maternal deaths were caused by complications after abortion, this proportion decreased to 69% in 1990. The decline continued steadily in subsequent years, concomitantly with improved access to safe abortion and modern family planning methods. Overall maternal mortality also showed a considerable decline, suggesting that the majority of maternal deaths prior to legislative reform resulted from unsafe abortions. Thus, in 2006, the overall maternal mortality rate was 15 deaths per 100,000 live births, while abortion-related mortality decreased to only 5 deaths per 100,000 births [9].

Contemporary national data on abortion-service availability, provision in the private sector, contraceptive access, and post-abortion outcomes remain incomplete in Romania, limiting precise assessment of current access patterns and regional disparities.

### 3.2. Epidemiology and Health Outcomes

Unsafe abortion, together with the lack of appropriate medical support in the post-abortion period, continues to represent a major challenge for global public health, contributing substantially to maternal mortality. The latter remains one of the leading causes of death among women of reproductive age, particularly in developing countries, where access to safe medical services is limited and the legislative framework regarding abortion is extremely restrictive.

According to estimates, approximately 21.6 million unsafe abortions were recorded in 2008, most of them in countries where legislation prohibits or severely restricts access to voluntary termination on request. These abortions have dramatic consequences for women’s health: approximately 47,000 women die each year from complications resulting from these interventions, corresponding to a rate of 220 deaths per 100,000 unsafe abortions [20]. These figures are alarming, given that they account for approximately 13% of total maternal mortality worldwide, with even higher percentages in certain disadvantaged regions, where this indicator may reach as high as 49% [20,21] (Table 1).

**Table 1:** Global and Romanian indicators related to induced abortion and maternal health.

Indicator	Global Context	Romanian Historical Context	Sources
Induced abortion incidence	Approximately one in four pregnancies ended in induced abortion worldwide during 1990–2019.	Incidence was exceptionally high after legalization; in 1991, 789,096 legal induced abortions were recorded, compared with approximately 270,000 births.	[1,3,10,12,13,15,16]
Unsafe abortion and mortality	Unsafe abortion remains concentrated in settings with restrictive laws and limited access to safe services.	Under restrictive pronatalist policy, unsafe abortion was a major contributor to maternal mortality; abortion-related mortality fell from 148 per 100,000 live births in 1989 to 58 in 1990 and 5 in 2006.	[4,6,11,20,21,22]
Maternal morbidity	Unsafe abortion contributes substantially to preventable morbidity, particularly where safe services are inaccessible.	The burden of complications was high before 1989 and declined during the 1990s and 2000s alongside legal access, safer care, and expanded family-planning services.	[3,4,13,15,16,20,22]
Contraception and family planning	Contraceptive access is a central component of preventing unintended pregnancy and abortion-related harm.	Modern contraceptive use increased from 13.9% in 1993 to 38.2% in 2004; the abortion rate subsequently declined to 27.1 per 1000 women in 2006.	[3,11,12,15,16,17,18,19]

Global and Romanian figures originate from different data sources, periods, and measurement approaches; they are presented to provide context and should not be interpreted as directly comparable estimates.

Moreover, the impact of unsafe abortions is not limited to fatal outcomes. Approximately 5 million women in low- and middle-income countries are hospitalized each year because of severe complications such as hemorrhage, infection, or uterine injury, all of which require costly treatment and sometimes surgical intervention. In many cases, these complications lead to long-term sequelae affecting fertility, mental health, and the quality of life of the affected women [22].

These data underscore the urgency of adopting systemic measures to prevent unsafe abortions through the promotion of universal access to effective contraceptive methods, comprehensive sexual education, and safe and legal reproductive health services. At the same time, restrictive abortion policies need to be revised, since they do not reduce the number of pregnancy terminations but merely push them into the informal and dangerous area of the medical system, placing women’s lives at risk. Thus, ensuring a favorable environment in which women can make informed and safe decisions regarding their reproductive health represents a fundamental objective for protecting their rights and lives.

More recent modeling work by Bearak et al. indicates that unintended pregnancy and abortion are markedly concentrated among women in low- and middle-income settings and in countries with restrictive legal frameworks, reinforcing the association between legal status, health-system barriers, and the use of unsafe or clandestine procedures [1].

The 2021 study by Clarke and Mühlrad investigates the impact of abortion law reforms on women’s health in Mexico, providing a rigorous analysis based on nationwide administrative data [23]. The study explores the effects of both liberalization and the tightening of abortion laws, offering population-level evidence on how legal changes influence maternal morbidity and mortality [23].

In 2007, the Federal District (Mexico City) adopted a major reform—known as ILE (Interrupción Legal del Embarazo)—which legalized and subsidized elective first-trimester abortion [24]. By contrast, numerous other Mexican states responded with regressive laws, reinforcing restrictions and increasing penalties for illegal abortion [25]. This internal legislative diversity provided an ideal framework for evaluating the differential impact of these policies on women’s health.

The study uses more than 46 million hospitalizations, 30 million births, and 18,400 maternal deaths recorded during the 2001–2015 period. The authors apply difference-in-differences and event-study econometric models, controlling for temporal and regional variation as well as confounding variables, in order to isolate the effects of legislative reforms on health outcomes.

The results show that legalization of abortion in Mexico City led to:

- an approximately 20% reduction in abortion-related morbidity;
- a 35% reduction in hemorrhage in early pregnancy;
- an 8% decline in fertility as a secondary effect;
- and a downward trend in maternal mortality, although the estimates for mortality were less precise due to the rarity of the phenomenon [23].

By contrast, the tightening of abortion laws in other Mexican states did not have a significant impact on maternal morbidity or mortality. Although some reforms constitutionally declared that life begins at conception, these were not accompanied by an observable reduction in the incidence of maternal complications.

The authors identify three possible channels through which legislative reform may influence women’s health:

1. quality of care—legalization of abortion allows procedures to be moved from unsafe environments to safe ones, thereby reducing risks;
2. reduction in unintended pregnancies—women may safely terminate pregnancies that pose health risks;
3. change in the composition of the maternal population—women who carry pregnancies to term are, on average, better prepared and healthier [23].

Furthermore, the article emphasizes that analyzing maternal mortality alone may seriously underestimate the real impact of reproductive health policies. Maternal morbidity, which is far more frequent, provides a broader and more relevant picture of women’s health [23].

The study provides strong empirical evidence that legal access to safe abortion contributes substantially to improving maternal health, without adverse effects on other dimensions of public health. The ILE reform in Mexico City is presented as an example of best practice for other countries undergoing revision of legislation concerning reproductive rights.

Beyond Latin America, quasi-experimental and health economics studies from Europe provide additional evidence that reforms improving legal status and access can reduce abortion incidence and complications. In Italy, Pieroni et al. used administrative data and a difference-in-differences design to show that the 2007 EU enlargement, which granted legal status and citizenship rights to Romanian and Bulgarian immigrant women, was associated with a 60–70% reduction in voluntary pregnancy terminations among this group, suggesting that secure legal status and social integration can substantially influence reproductive behavior [26]. This finding is consistent with broader international evidence suggesting that legal status and access to reproductive-health services may influence reproductive decision-making and health outcomes [23,26].

### 3.3. Sociodemographic and Psychosocial Determinants

Available evidence suggests that the decision to resort to abortion is influenced by economic factors, psychosocial conditions, and the inefficient or inconsistent use of contraceptive methods. A low level of education regarding sexual health may favor the occurrence of unintended pregnancies, thereby contributing to an increased number of induced abortions on request.

Given the complex historical context of abortion in Romania, marked by severe legislative restrictions during the communist period and rapid liberalization after 1989, it is essential at present to focus on the factors that lead women to resort to induced abortion in the twenty-first century. Despite advances in medical care, access to information, and the availability of contraceptive methods, induced abortion on request continues to be perceived by some women as a feasible solution when faced with an unintended pregnancy. This choice is rarely simple or superficial, being influenced by a complex set of personal, economic, educational, and psychosocial factors.

A systematic review of the literature published in 2009 synthesized the conclusions of several studies on the motivations for induced abortion, proposing a conceptual classification into four major categories: woman-focused reasons, other-focused reasons, material reasons, and complex or contingent reasons [27].

In the first category, namely woman-focused reasons, the most frequently cited motive was that of inappropriate timing—“wrong timing”—in the sense that the woman wants a child, but not at that point in her life. Other justifications in this category include concerns related to physical or mental health, the fact that the woman already has a considerable number of children, the lack of desire to have children, or fear of giving birth to a child with disabilities [27].

The category of other-focused reasons includes a variety of situations. Among these, the relationship with the partner plays an essential role: his absence, relationship instability, or the fact that it is a recent relationship were frequently mentioned. Reasons related to the unborn child are also included, such as the lack of desire to have that child, the perception of unfavorable conditions for raising the child, or fear of congenital anomalies. Other influences come from third parties, such as family or community, reflecting ethnic, religious, or social pressures, as in situations where the woman is considered too young to become a mother. This category also includes cases in which the existence of previous children leads the woman to consider the pregnancy an unsustainable burden, especially in the context of suspected fetal malformations. In addition, although less frequently mentioned in the literature reviewed, cases of domestic violence, sexual abuse, rape, or incest are also described [27].

The third category brings together material reasons, particularly those referring to financial instability or the lack of permanent housing—factors that directly affect the perception of the ability to sustain a pregnancy and raise a child [27].

Finally, the category of complex and contingent reasons includes those justifications in which the woman anticipates an irreversible impact of childbirth on her own life course. These include interference with education, professional career, or responsibilities related to caring for other dependents. These reasons underline the complexity of the decision to terminate a pregnancy, in which personal plans and social responsibilities intersect in a profound and often difficult-to-manage way [27]. These patterns are summarized in Table 2.

Population-based analyses add granularity to these conceptual categories. In Spain, Baranda Ortiz and Stanek showed that pregnancies ending in induced abortion are disproportionately concentrated among young women without a partner, who use abortion to avoid early motherhood and disruption of education and labor market entry, and among older women with two or more children, who resort to abortion primarily to limit or end childbearing [28]. The probability of induced abortion was also higher among women with lower educational attainment and those outside the labor market, confirming the central role of socioeconomic vulnerability across age groups.

These findings agree with global model estimates indicating that unintended pregnancy and abortion are more frequent in settings with restrictive laws, low contraceptive coverage, and high income inequality [1]. They also converge with qualitative evidence showing that women’s decisions are often shaped by anticipated educational, employment, and caregiving trajectories, rather than isolated, short-term preferences [27,29].

In addition to individual and structural factors, provider attitudes and conscientious objection may influence when and how women can act on their reproductive decisions. In Italy, high levels of conscientious objection have been documented among healthcare professionals, with an estimated 64.6% of gynecologists, 44.6% of anesthetists, and 36.2% of other staff declaring themselves objectors [30]. More recent survey data suggest that objection status may change under specific conditions, such as the involvement of multi-collegiate commissions or cases of serious maternal or fetal illness and sexual violence [31].

This interpretive framework invites a reconsideration of reproductive health policies, with prevention—through sexual education, access to contraception, and social support—placed at the center of concern. There is also a need for a nonjudgmental approach centered on the woman’s experience and respect for her autonomy, rather than on moralizing or punitive discourse. Thus, understanding the real reasons behind the decision to terminate a pregnancy is an essential step in developing effective and equitable public health policies.

**Table 2:** Main categories of reasons and determinants for induced abortion on request.

Category	Typical Reasons/Determinants	Empirical Examples	Key Sources
Woman-focused reasons	Pregnancy at an unsuitable time, no desire for additional children, concerns about physical or mental health, parity and desired family size	Delaying childbearing, avoiding early motherhood, or limiting childbearing after reaching desired parity	[1,27,28]
Other-focused reasons	Partner absence or relationship instability, family or community pressure, fetal anomaly, violence or sexual abuse	Avoiding single parenthood; abortions following fetal-anomaly diagnosis; decisions influenced by partner or family expectations	[24,27,28]
Material reasons	Financial insecurity, unemployment, precarious housing, low educational attainment	Higher likelihood among women with lower education or outside the labor market; pregnancy perceived as unaffordable	[1,27,28]
Complex/contingent reasons	Anticipated effect on education, employment, caregiving responsibilities, migration projects	Concern about interruption of studies; limiting childbearing to maintain employment or care for dependents	[23,27,28]
Structural and system-level factors	Legal and migration status, availability and geographic distribution of services, conscientious objection, stigma	Decline in induced abortions after improved legal status; access difficulties where conscientious objection is common; delays or avoidance of care due to stigma	[23,26,29,30,31]

### 3.4. Psychological Outcomes and Counseling

Most evidence on psychological and psychosexual outcomes identified in this review originates from countries other than Romania. These studies provide comparative context, but differences in healthcare organization, stigma, social support, legal frameworks, and participant characteristics may limit their direct applicability to Romanian women.

The longitudinal study published by Di Febo et al. in 2018 compared women undergoing induced abortion on request—classified by the authors as voluntary termination of pregnancy (VTP)—with women undergoing therapeutic termination of pregnancy (TTP) [32]. The central objective of the research was to identify risk predictors for the development of a negative post-abortion psychological response, based on personal, situational, and systemic characteristics [32].

In a sample of 92 women recruited between 2013 and 2015 at Policlinico Umberto I in Rome, two subgroups were formed: 57 women in the VTP group and 35 in the TTP group. Psychological assessments were carried out using two validated psychometric instruments—Symptom Checklist-90-Revised (SCL-90-R) and Center for Epidemiologic Studies Depression Scale (CES-D)—administered 5–7 days before and approximately 90 days after the pregnancy termination procedure. The statistical analyses (paired-samples *t* test and repeated-measures ANOVA) examined score variation over time and the influence of contextual factors such as partner support and the nature of the reason for abortion [32].

The results indicated a significant decrease in psychological symptoms in the VTP group, particularly in the dimensions of depression (DEP), anxiety (ANX), and somatization (SOM), in both the SCL-90-R and CES-D. This trend was interpreted as indicative of improvement in affective status in the post-abortion period, suggesting a reduction in emotional distress and internalizing symptoms, especially in women who benefited from emotional support from their partner (F_(1, 18)_ = 5.10, *p* = 0.036). Lack of partner support proved to be a significant predictor of a less favorable psychological outcome. It was also observed that women who terminated pregnancy for non-relational reasons, such as financial reasons or career concerns, showed a more pronounced decrease in depressive symptoms compared with those who cited relationship-related reasons (F_(1, 18)_ = 7.83; *p* = 0.012) [32].

With regard to the TTP group, although no significant changes were observed in global scores, significant decreases were identified in the somatization and anxiety subscales (SOM *p* = 0.023; ANX *p* = 0.016), as well as in the total CES-D score (*p* = 0.020). An interesting result was identified according to the type of fetal anomaly: women who terminated pregnancy following a diagnosis of trisomy 21 exhibited a higher degree of sleep disturbances, hostility, and obsessive-compulsive symptoms compared with those carrying a fetus with malformations incompatible with life (F_(1, 28)_ = 5.56; *p* = 0.026) [32].

These findings should be interpreted in the context of substantial heterogeneity in psychological responses following abortion. Available evidence does not support a uniform psychological trajectory: some women report relief or decreasing distress, whereas others may experience persistent distress, regret, grief, anxiety, depressive symptoms, or other clinically relevant difficulties. Post-abortion psychological outcomes appear to be influenced by personal, social, and cultural context, including pre-existing mental-health status, pregnancy intention and circumstances, relationship and social support, perceived stigma, coping strategies, attachment to the pregnancy, and decisional autonomy [33,34,35,36,37].

The study has several limitations, including a high dropout rate in the VTP group, with a reduction from 57 to 20 participants, and the lack of a control group. Nevertheless, the longitudinal methodology, the use of validated instruments, and the timing of the initial assessment, conducted during the abortion request phase rather than immediately before the procedure, are considered strengths of the study.

The available data suggest that abortion is not uniformly associated with deterioration in mental health; however, psychological outcomes vary across individuals and clinical circumstances. Pre-existing mental-health status, pregnancy intention, the reasons and circumstances surrounding the abortion, partner and social support, stigma, and decisional autonomy may influence emotional adjustment. These considerations support individualized, nonjudgmental counseling and appropriate psychological support when indicated [33,34].

A multicenter Italian study by Limoncin et al. examined psycho-sexological outcomes after first-trimester induced abortion on request, termed voluntary termination of pregnancy on request (VTOP) by the authors. In a sample of 170 women assessed at the time of abortion and six months later, the authors found significant decreases in depressive and anxiety symptoms and a modest improvement in overall sexual function [38]. However, nearly 70% of participants still met criteria for sexual dysfunction at follow-up, and younger women (18–25 years) exhibited less improvement than older age groups, suggesting age-related differences in vulnerability and adaptation [38].

Major empirical studies and two comprehensive reviews—the American Psychological Association (APA) task force report and the National Collaborating Centre for Mental Health (NCCMH) systematic review—conclude that induced abortion is not, in itself, a major determinant of long-term mental health problems, and that increased risk is associated primarily with pre-existing psychological difficulties, poor social support, and exposure to stigma rather than with the procedure per se [33,34,35,36,37].

The article published by Crandell in 2012 in Nursing for Women’s Health comparatively analyzes the psychological outcomes of first-trimester elective abortion using medical methods, based on mifepristone and misoprostol, and surgical methods, namely vacuum aspiration [39,40]. The main aim of the study was to understand the psychological impact of the two types of intervention on patients, in a context in which almost half of pregnancies in the United States are unplanned and 3 out of 10 women will have an abortion by the age of 45 [39,40].

The methodology involved a systematic review of the scientific literature from databases such as CINAHL, PubMed, and MEDLINE. Only studies published after 2003 that analyzed psychological outcomes in abortions performed up to a maximum gestational age of 14 weeks were included. Six relevant studies were selected, assessing variables such as anxiety, depression, post-traumatic stress, quality of life, and self-esteem, using validated instruments such as the Hospital Anxiety and Depression Scale (HADS), Edinburgh Postnatal Depression Scale (EPDS), Impact of Event Scale (IES), and European Organisation for Research and Treatment of Cancer Quality of Life Questionnaire Core 30 (EORTC QLQ-C30) [39,40].

The results indicated that, regardless of the method used, patients’ psychological condition improved significantly after the procedure [39]. For example, in the study by Hemmerling et al., levels of anxiety and depression decreased considerably at 4 weeks after abortion in both the medical and surgical groups [41]. Similarly, Ashok et al. found an important reduction in psychological symptoms 2–3 weeks after the intervention [42]. Crandell emphasizes that this pattern of decreased psychological distress is supported by all reviewed studies except that of Yilmaz et al., which did not include a pre-procedure assessment and found a higher prevalence of depression in the surgical group (34.3% vs. 22.7%) [43].

Although the differences between methods are generally minimal, some studies identified slightly higher levels of anxiety or depression among patients who underwent surgical abortion. For example, in Hemmerling et al., 39.1% of patients in the surgical group had clinically significant anxiety before the procedure, compared with 27.2% in the medical group. At 4 weeks after the intervention, these percentages had declined to 6.3% and 3.7%, respectively [41]. In addition, the study by Yilmaz et al. reported significantly higher EPDS scores in patients who underwent surgical abortion [43].

Regarding post-traumatic stress, the results are less conclusive. For example, Hemmerling et al. reported higher scores on the intrusion subscale in the surgical group (*p* = 0.039), suggesting a greater likelihood of intrusive thoughts related to the event [41]. By contrast, Robson et al. found higher scores among patients who had medical abortions, especially in the randomly allocated groups [44].

Another noteworthy aspect is the impact of choosing the abortion method on psychological status. Studies indicate that women who were able to choose their preferred method generally had better psychological outcomes than those who were randomly assigned [42,44]. This suggests that freedom of choice may act as a protective factor against the emotional impact of the procedure.

The psychological outcomes following first-trimester elective abortion are generally positive, regardless of whether the method used is medical or surgical. Differences between methods are small, while factors such as gestational age, previous mental health experiences, and decisional autonomy appear to play a significant role in psychological outcomes. The author recommends that healthcare professionals take these data into account in patient counseling, emphasizing balanced information and individualized support [39]. The main characteristics and findings of these studies are summarized in Table 3.

**Table 3:** Selected studies on psychological and psycho-sexual outcomes after induced abortion.

Study (Country)	Design and Sample	Follow-Up	Main Findings	Key Sources
Adler et al., USA	Prospective cohort of women undergoing first-trimester abortion and women carrying unintended pregnancies to term	Up to 2 years	Short-term distress but not with long-term psychopathology; prior mental health and contextual factors more influential than abortion itself	[37]
Major et al., USA	Prospective study of women after first-trimester induced abortion	Multiple assessments over 2 years	Distress generally decreased and decision satisfaction was high; prior mental-health problems predicted poorer outcomes	[35]
Steinberg & Russo, USA	Secondary analysis of large cohort	Not specified in the summary	No evidence of increased long-term anxiety after accounting for pre-existing anxiety and other risk factors	[36]
APA Task Force; NCCMH, USA/UK	Narrative review and systematic review	Studies with varied follow-up periods	Induced abortion was not independently associated with mental illness; outcomes were more strongly associated with prior mental health, social circumstances, and stigma	[33,34]
Di Febo et al., Italy	Longitudinal study; 92 women (57 VTP; 35 TTP)	Baseline and approximately 90 days after termination	Depression, anxiety, and somatization decreased after VTP; partner support was associated with better adjustment	[32]
Limoncin et al., Italy	Multicenter observational study; 170 women undergoing first-trimester VTOP	Baseline and 6 months	Depression and anxiety decreased and sexual function scores improved modestly; younger participants showed less improvement	[38]
Crandell, USA	Review comparing medical and surgical first-trimester induced abortion	Several weeks after procedure	Psychological outcomes improved with both methods; differences were small, while autonomy and gestational age appeared more relevant	[39]
Hemmerling et al., Germany	Prospective study of medical abortion with mifepristone	Short-term follow-up	High acceptability and generally transient emotional distress; most participants reported relief	[41]
Ashok et al., UK	Comparative study of medical vs. surgical abortion at 10–13 weeks	Post-procedure follow-up	Distress decreased in both groups; method choice and counseling quality influenced responses	[42]
Yilmaz et al., Turkey	Prospective comparison of medical vs. surgical abortion	Short-term follow-up	No major long-term psychiatric differences between methods; early distress generally resolved	[43]
Robson et al., UK	Randomized preference trial of medical vs. surgical abortion at ≤14 weeks	Post-procedure follow-up	Ability to choose the preferred method was associated with greater satisfaction and better psychological outcomes	[44]

Abbreviations: APA, American Psychological Association; NCCMH, National Collaborating Centre for Mental Health; TTP, therapeutic termination of pregnancy; VTOP, voluntary termination of pregnancy on request; VTP, voluntary termination of pregnancy.

### 3.5. Language and Terminology in Abortion Care

The article published by Janiak and Goldberg in 2016 analyzes the linguistic, clinical, and ethical implications of using the expression “elective abortion” in medical and public discourse. It is argued that this term fails to reflect the complexity of the decision to terminate a pregnancy and contributes to perpetuating stigma surrounding abortion. The frequently used distinction between “elective abortion” and “indicated abortion” is considered arbitrary, difficult to apply consistently, and productive of false hierarchies among patients [45].

In medical practice, the term “elective” is used to designate interventions that are not urgent; however, this does not imply the absence of a real need. Thus, equating abortion with an optional or superficial procedure ignores the multitude of medical, psychological, economic, and social factors that influence the decision to terminate a pregnancy. In addition, the history of the use of the term shows that access to abortion was previously conditioned by the approval of medical committees in a way that favored women with greater resources and higher socioeconomic status, to the detriment of marginalized women.

By establishing a separation between “justified” abortions, for example, in cases of medical risk or malformations, and “elective” abortions motivated by personal or social reasons, a moral hierarchy of reproductive motivations is perpetuated. There is no clear consensus, either among healthcare professionals or in the public sphere, on what constitutes a “valid” indication for abortion. In many situations, the risks perceived by the patient—whether related to mental health, economic security, personal relationships, or educational trajectories—are difficult to fit into a rigid classification.

The expression “elective abortion” is semantically loaded with an implicit value judgment, contributing to the stigmatization of women who choose this procedure. The cited studies show that some individuals who underwent abortion following genetic or medical diagnoses prefer to avoid the term “abortion” precisely in order to distance themselves from the negative connotations associated with it [29].

Instead, the use of the term “induced abortion” is proposed to designate any voluntary termination of pregnancy, distinct from “spontaneous abortion.” Where necessary, clinical or contextual details may be added, but without resorting to terms that imply moral hierarchies or stigma. Maintaining the expression “elective abortion” in medical and scientific vocabulary is not justified, since it does not contribute to a better understanding of the phenomenon, but instead diminishes the value of patients’ experiences and compromises the neutrality of professional language [45].

The Italian debate on conscientious objection and hyper-regulation of abortion care also illustrates how legal language and institutional framing can influence both provider behavior and women’s experiences. Analyses of national law and recent surveys show that strict procedural requirements, combined with high rates of conscientious objection, may symbolically reinforce a perception of abortion as exceptional or morally suspect, despite formal legality [31,45].

Conceptualizing abortion stigma as arising from social, cultural, and institutional narratives rather than from the procedure itself suggests that clinical and scientific language should avoid categories that implicitly rank abortions as “justified” or “elective” and instead focus on precise, non-hierarchical descriptors and the broader context of women’s lives [29,45].

## 4. Discussion

The literature reviewed indicates that induced abortion is closely intertwined with historical, legislative, and socioeconomic conditions rather than being a purely individual choice [1,3,4,5,11,23]. In Romania, the transition from highly restrictive pronatalist policies to legal access to abortion and expanded family planning services was temporally associated with substantial changes in abortion patterns and maternal health indicators. These trends are consistent with the potential influence of legislation, health-system organization, contraceptive availability, and healthcare quality, although they may also reflect broader social, economic, demographic, and reporting-related changes [2,4,6,7,11,12,13,14,15,16,17,18,19]. At the same time, the persistence of abortion as a family planning strategy in some settings reflects structural vulnerabilities related to poverty, unequal access to contraception, limited sexual education, and unstable partner or family circumstances [3,12,15,16,18,19,24,27,29]. These domains—historical context, health-system organization, sociodemographic conditions, psychological outcomes, and language—are synthesized in Table 4 to highlight their implications for clinical practice and policy.

Romania shares with other restrictive settings the association between limited contraceptive access, unsafe abortion, and adverse maternal-health outcomes. However, its experience differs in several important respects. The exceptionally restrictive pronatalist policies of the communist period, the abrupt legalization of abortion after 1989, and the subsequent expansion of family-planning services created a rapid and historically distinctive transition from clandestine and unsafe procedures to legal care. In contrast, evidence from Mexico and other settings often evaluates more geographically limited or incremental legal reforms within different health-system and social contexts. Across countries, the literature consistently supports the importance of safe service provision, contraceptive access, and social conditions in shaping reproductive-health outcomes. However, uncertainty remains regarding the relative contribution of legal reform, health-system improvements, socioeconomic change, demographic shifts, and changes in reporting practices to observed trends. These differences limit direct comparison across settings and preclude simple causal attribution of Romanian trends to any single policy intervention.

The evidence synthesized in this review is subject to several important limitations that should be considered when interpreting its implications. In addition, because this narrative review integrated heterogeneous source types and did not include a formal risk-of-bias assessment or certainty-of-evidence grading, the relative methodological strength of individual sources could not be compared systematically. Much of the information on maternal mortality and morbidity related to unsafe abortion comes from observational and ecological studies relying on administrative or aggregated data, which may under-report events or mask regional and socioeconomic disparities [8,9,20,21,22,23]. In Romania, estimates of abortion incidence and abortion-related deaths before and after legislative reforms are derived from health system statistics and retrospective analyses, often without standardized reporting across facilities or consistent definitions of unsafe procedures [3,8,9,10,11,12,13,15,16]. Contemporary Romanian evidence is further limited by incomplete routine reporting across public and private providers and by the scarcity of nationally representative information on abortion-service availability, contraceptive access, post-abortion outcomes, and women’s experiences of care. Many studies examining psychosocial determinants and psychological outcomes after abortion are based on relatively small samples, single-center recruitment, self-report instruments, and incomplete follow-up, which limits the generalizability of their findings and raises concerns about attrition bias, selection bias, residual confounding, and reverse causation [32,33,34,35,36,37,39,41,42,43,44]. These methodological constraints do not invalidate the overall patterns observed—particularly the association between restrictive policy environments, unsafe abortion, and maternal health outcomes, and the importance of social support and decisional autonomy for psychological well-being—but they do call for cautious interpretation, preclude causal inference from observed temporal trends, and highlight the need for more robust prospective data [8,20,21,22,23,32,33,34,35,36,37,39].

Taken together, the available data suggest that the consequences of abortion policy extend well beyond the formal legality of the procedure. Evidence from Romania and other international settings suggests that greater access to safe abortion care, post-abortion counseling, and contraception is associated with lower abortion-related morbidity and mortality, whereas restrictive legal and service environments are associated with a greater burden of unsafe practices and preventable complications [4,8,9,11,20,21,22,23]. These associations should be interpreted cautiously because much of the available evidence is observational or ecological. The psychological literature reviewed indicates substantial heterogeneity in post-abortion experiences. Although abortion is not uniformly associated with adverse long-term mental-health outcomes, emotional adjustment may vary according to pre-existing mental-health status, pregnancy intention and circumstances, partner and social support, stigma, coping resources, and decisional autonomy [32,33,34,35,36,39,41,42,44].

From a clinical and public health perspective, these findings support a patient-centered and nonjudgmental approach to abortion care. Counseling should be grounded in balanced information, respect for autonomy, and awareness of the multiple determinants that influence reproductive decisions [32,33,34,35,36,39,41,42,44]. In parallel, the language used in both scientific writing and clinical communication remains highly relevant, because stigmatizing terminology may distort professional neutrality and diminish the lived experience of patients [29,45]. Using precise, non-hierarchical terms such as “induced abortion” and avoiding subjective categorizations can help align clinical communication with the ethical principles of respect and beneficence [29,45].

Important evidence gaps remain in contemporary Romanian data on reproductive-health service provision, regional inequities, and women’s experiences of care. Nationally coordinated monitoring of abortion-service availability, private-sector provision, contraceptive access, and abortion-related morbidity would improve understanding of current needs and disparities. Prospective longitudinal studies of post-abortion psychological outcomes, including stigma, partner support, pregnancy intention, and counseling, are also needed to establish how far international evidence applies within Romanian cultural and health-system settings [29,32,33,34,35,36,37,39]. Mixed-methods research combining quantitative data on abortion incidence, contraceptive use, and maternal morbidity with qualitative interviews exploring women’s decision-making processes could provide a more nuanced understanding of abortion as a family planning strategy [3,12,15,16,18,19,23,27,29]. Strengthening routine reporting systems for induced abortion and post-abortion complications, ideally integrated with contraceptive and reproductive health indicators, would improve the capacity to monitor trends and evaluate interventions [3,8,9,10,12,13,15,16,20]. From a policy perspective, improving equitable access to modern contraception, high-quality counseling, and safe abortion services, together with training of healthcare professionals in nonjudgmental communication and non-stigmatizing terminology, may help reduce preventable maternal morbidity and support women’s reproductive autonomy [3,15,16,17,20,21,22,23,29,45]. Implementing and rigorously evaluating post-abortion and postpartum family planning programs in obstetrics and gynecology services could represent a concrete step toward translating the evidence synthesized in this review into practice [3,11,15,16,17,18,19,23].

**Table 4:** Key implications for practice and policy.

Domain	Condensed Implications	Main Sources
Historical and legislative context	Restrictive policy and limited contraception were associated with unsafe abortion and maternal mortality in Romania.	[3,4,5,6,8,9,11,13,15,16]
Health system and access	Safe services, counseling, and contraception should be accessible and equitable.	[3,11,13,15,16,17,23,30,31]
Sociodemographic and life-course factors	Counseling should recognize economic, relational, and life-course circumstances.	[1,23,24,26,27,28]
Psychological and psychosexual outcomes	Assess support needs and provide individualized, nonjudgmental care.	[32,33,34,35,36,37,38,39,41,42,43,44]
Language, stigma and communication	Use neutral terminology and avoid language that moralizes reproductive decisions.	[29,30,31,45]

## 5. Conclusions

Induced abortion represents a complex phenomenon situated at the intersection of medical, social, economic, legal, and cultural factors. In the Romanian context, recent history shows an evolution marked by legislative transitions and profound changes in access to reproductive health services. From the restrictive pronatalist policies of the communist period to the abrupt liberalization after 1989, abortion was perceived and used, in many cases, as the main method of family planning.

Modernization of the healthcare system expanded access to contraceptives, and development of counseling services was accompanied by substantial declines in reported abortion incidence and abortion-related maternal mortality; however, these temporal patterns cannot establish causality and may also reflect broader social, economic, demographic, and reporting-related changes.

From a psychological perspective, induced abortion is not automatically associated with negative effects on mental health. For some women, it may represent a rational and relieving response to an unintended pregnancy, particularly when care is provided safely, with emotional support, and on the basis of an informed decision. Post-abortion psychological adjustment may be influenced by the patient’s personal context, prior mental-health status, pregnancy circumstances, social support, and the quality of the relationship with medical staff.

Approaching abortion as an integral component of public health policy requires an empathetic and nonjudgmental perspective. National policies should support women’s reproductive autonomy and seek to facilitate access to sexual education, modern contraceptive methods, and safe, respectful reproductive-health services within an appropriate legal and clinical framework.

In light of the above, a patient-centered approach to reproductive health remains essential, with emphasis on women’s real experiences and on interventions based on individual needs and respect for fundamental rights.

## Figures and Tables

**Figure 1:**
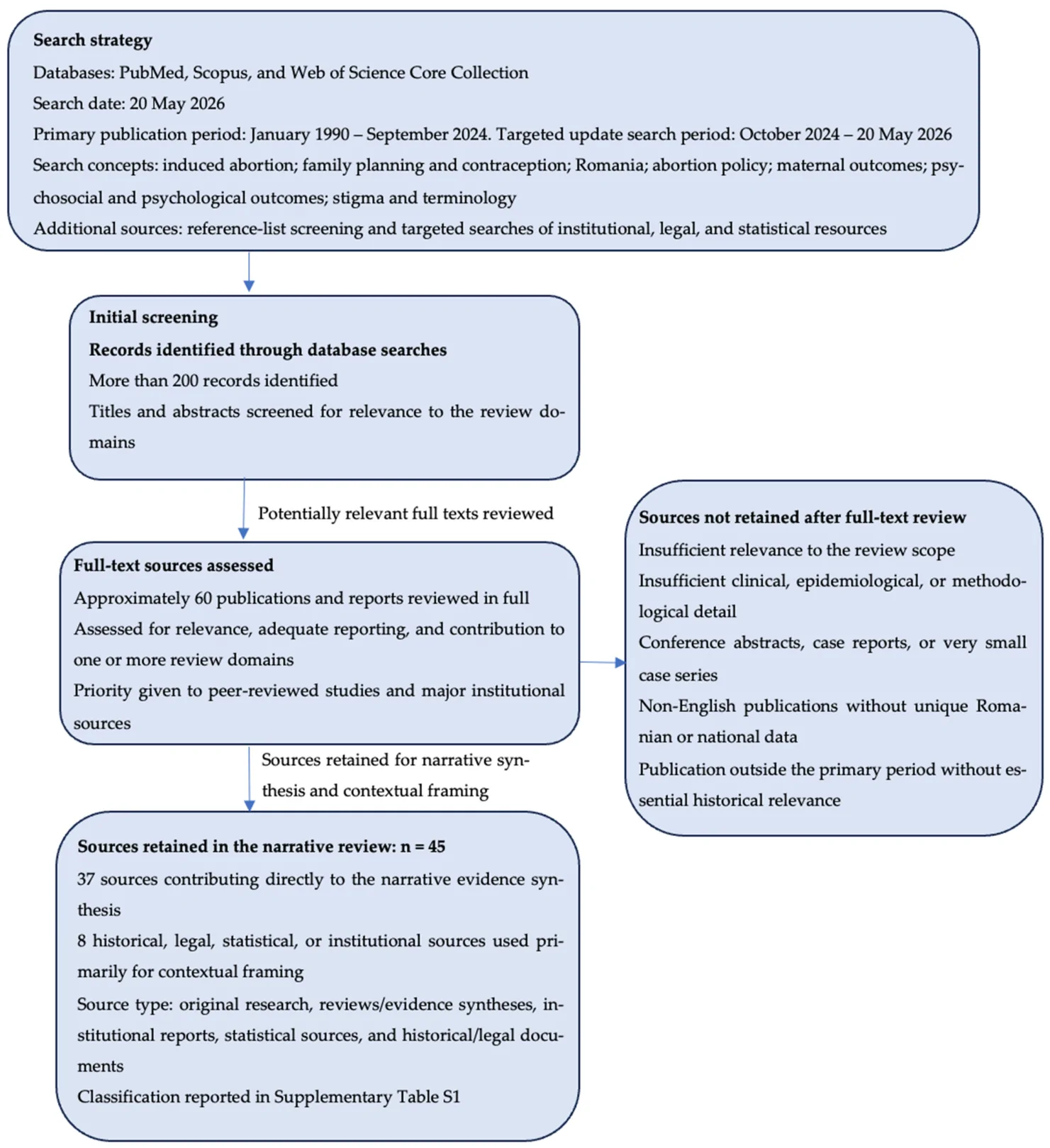
Structured identification and selection of sources for the narrative literature review. The figure summarizes the database search, relevance screening, full-text review, and retention of empirical, institutional, statistical, historical, and legal sources.

## Data Availability

No new data were created or analyzed in this study. Data sharing is not applicable to this article.

## Supplementary Materials

The following supporting information can be downloaded at: https://www.mdpi.com/article/10.3390/healthcare14203372/s1, Table S1: Classification and role of the 45 sources cited in the narrative review; Table S2: Database-specific electronic search strategy.

## Abbreviations

The following abbreviations are used in this manuscript:

ANOVA	Analysis of variance
ANX	Anxiety
APA	American Psychological Association
BDI-II	Beck Depression Inventory–II
CDC	Centers for Disease Control and Prevention
CES-D	Centre for Epidemiologic Studies Depression Scale
DEP	Depression
EORTC QLQ-C30	European Organisation for Research and Treatment of Cancer Quality of Life Questionnaire Core 30
EPDS	Edinburgh Postnatal Depression Scale
EU	European Union
FSFI	Female Sexual Function Index
HADS	Hospital Anxiety and Depression Scale
IES	Impact of Event Scale
ILE	Interrupción Legal del Embarazo (Legal Termination of Pregnancy)
INSSE	National Institute of Statistics, Romania (Institutul Național de Statistică)
ISI	Institute for Scientific Information
MOH	Ministry of Health
NCCMH	National Collaborating Centre for Mental Health
NFPP	National Family Planning Program
PSI	Population Services International
RFHI	Romanian Family Health Initiative
SAS	Self-Rating Anxiety Scale
SCL-90-R	Symptom Checklist-90-Revised
SOM	Somatization
TTP	Therapeutic termination of pregnancy
USAID	United States Agency for International Development
VTOP	Voluntary termination of pregnancy
VTP	Voluntary termination of pregnancy
WHO	World Health Organization
