# Supplementary material for: Induced Abortion as a Family Planning Strategy in Romania: A Narrative Literature Review of Policy, Health Outcomes and Psychosocial Determinants
Source: Healthcare (Basel). 2026 Oct 9;14(20):3372. doi: 10.3390/healthcare14203372 (PMC13704172; doi:10.3390/healthcare14203372)
Supplement: Supplementary file 1 [file healthcare-14-03372-s001.zip › healthcare-4586373-supplementary.pdf]

Supplementary Materials

Supplementary Table S1. Classification and role of the 45 sources cited in the narrative review.

Category	Reference numbers	Number	Role
Original empirical research articles	3, 4, 11, 13, 21–23, 26, 28, 30–32, 35–38, 41–44	20	Direct evidence synthesis
Review articles and evidence syntheses	1, 20, 24, 27, 34, 39, 45	7	Direct evidence synthesis
Institutional reports, program reports, and policy documents	8, 9, 14–19, 33, 40	10	Direct evidence synthesis and/or policy/program context
Statistical databases and yearbooks	10, 12	2	Historical and epidemiological context; descriptive trend data
Historical, legal, and contextual documents	2, 5–7, 17, 25	6	Historical, legal, and institutional framing
**Total**	**1–45**	**45**	**Narrative review sources**

Classification reflects the primary role of each source in the present narrative review. Some institutional reports and policy documents contributed both contextual information and descriptive epidemiological or program-related evidence.

Supplementary Table S2. Database-specific electronic search strategy.

Database	Date searched	Search fields	Search concepts and terms	Combination of concepts	Limits
PubMed/MEDLINE	20 May 2026	MeSH terms and title/abstract fields	**Abortion:** “induced abortion,” “voluntary termination of pregnancy,” “termination of pregnancy,” “elective abortion,” “unsafe abortion.” **Family planning:** “family planning,” contraception, contraceptive use, “post-abortion contraception.” **Romanian context:** Romania, Romanian. **Policy and health outcomes:** “abortion law,” “abortion policy,” legislation, “reproductive policy,” “maternal mortality,” “maternal morbidity.” **Psychosocial outcomes and language:** “mental health,” psychological, psychosocial, depression, anxiety, stigma, terminology, “conscientious objection.”	Abortion-related terms were combined with at least one additional concept group: family planning/contraception; Romania/Romanian context; policy, legislation, or maternal outcomes; psychological or psychosocial outcomes; or stigma, terminology, and conscientious objection.	Publication date: 1 January 1990–30 September 2024; English language; human studies where applicable.
Scopus	20 May 2026	Title, abstract, and keywords	**Abortion:** “induced abortion,” “voluntary termination of pregnancy,” “termination of pregnancy,” “elective abortion,” “unsafe abortion.” **Family planning:** “family planning,” contraception, contraceptive use, “post-abortion	Abortion-related terms were combined with one or more additional concept groups according to the review domain: Romanian historical and policy context; epidemiology and	Publication date: 1 January 1990–30 September 2024; English language;

			contraception." **Romanian context:** Romania, Romanian. **Policy and health outcomes:** "abortion law," "abortion policy," legislation, "reproductive policy," "maternal mortality," "maternal morbidity." **Psychosocial outcomes and language:** "mental health," psychological, psychosocial, depression, anxiety, stigma, terminology, "conscientious objection."	maternal outcomes; determinants of abortion; psychological outcomes; or terminology and stigma.	articles and reviews, with relevant indexed documents considered where appropriate.
Web of Science Core Collection	20 May 2026	Topic field: title, abstract, author keywords, and Keywords Plus	**Abortion:** "induced abortion," "voluntary termination of pregnancy," "termination of pregnancy," "elective abortion," "unsafe abortion." **Family planning:** "family planning," contraception, contraceptive use, "post-abortion contraception." **Romanian context:** Romania, Romanian. **Policy and health outcomes:** "abortion law," "abortion policy," legislation, "reproductive policy," "maternal mortality," "maternal morbidity." **Psychosocial outcomes and language:** "mental health," psychological, psychosocial, depression, anxiety, stigma, terminology, "conscientious objection."	Abortion-related terms were combined with at least one thematic concept group. Search combinations were adapted to identify evidence relevant to the main domains of the narrative review rather than to answer a single narrowly defined clinical question.	Publication date: 1 January 1990–30 September 2024; English language; articles, reviews, and relevant indexed records.

Search terms within each concept group were combined using "OR," while the abortion-related concept group was combined with relevant thematic concept groups using "AND." The database searches were supplemented by reference-list screening of key reviews, institutional reports, policy documents, and relevant articles. Additional historical, legal, statistical, and institutional documents were identified through targeted searches of relevant governmental and organizational websites. No study-design filter was applied. The targeted update search was conducted to identify studies published after the end date of the primary search. Publications from 2025 and 2026 were considered for inclusion when they met the same relevance-based eligibility criteria applied to sources identified through the primary search.
